# Pannexin 1 Regulates Network Ensembles and Dendritic Spine Development in Cortical Neurons

**DOI:** 10.1523/ENEURO.0503-18.2019

**Published:** 2019-06-06

**Authors:** Juan C. Sanchez-Arias, Mei Liu, Catherine S. W. Choi, Sarah N. Ebert, Craig E. Brown, Leigh Anne Swayne

**Affiliations:** Division of Medical Sciences, University of Victoria, Victoria, British Columbia V8P 5C2, Canada

**Keywords:** cortical neuron, critical period, dendritic spines, network ensembles, pannexin, somatosensory

## Abstract

Dendritic spines are the postsynaptic targets of excitatory synaptic inputs that undergo extensive proliferation and maturation during the first postnatal month in mice. However, our understanding of the molecular mechanisms that regulate spines during this critical period is limited. Previous work has shown that pannexin 1 (Panx1) regulates neurite growth and synaptic plasticity. We therefore investigated the impact of global Panx1 KO on spontaneous cortical neuron activity using Ca^2+^ imaging and *in silico* network analysis. Panx1 KO increased both the number and size of spontaneous co-active cortical neuron network ensembles. To understand the basis for these findings, we investigated Panx1 expression in postnatal synaptosome preparations from early postnatal mouse cortex. Between 2 and 4 postnatal weeks, we observed a precipitous drop in cortical synaptosome protein levels of Panx1, suggesting it regulates synapse proliferation and/or maturation. At the same time points, we observed significant enrichment of the excitatory postsynaptic density proteins PSD-95, GluA1, and GluN2a in cortical synaptosomes from global Panx1 knock-out mice. *Ex vivo* analysis of pyramidal neuron structure in somatosensory cortex revealed a consistent increase in dendritic spine densities in both male and female Panx1 KO mice. Similar findings were observed in an excitatory neuron-specific Panx1 KO line (Emx1-Cre driven; Panx1 cKO^E^) and in primary Panx1 KO cortical neurons cultured *in vitro.* Altogether, our study suggests that Panx1 negatively regulates cortical dendritic spine development.

## Visual Abstract

**Figure:**
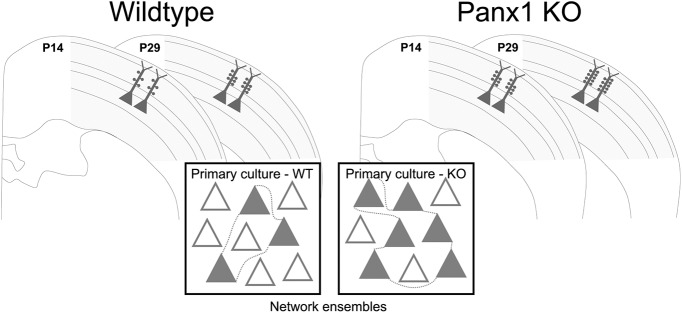

## Significance Statement

Our findings reveal an important regulatory role for pannexin 1 (Panx1) in the formation of connections between nerve cells. We found that removal of the Panx1 altered the ability of nerve cells from the cerebral cortex to fire together. We studied the impact of removing Panx1 on the formation of “dendritic spines”, which are microscopic protrusions that receive information from other nerve cells. We found that removing Panx1 increased the expression of proteins involved in dendritic spine function and also increased the density of dendritic spines on nerve cells of the cerebral cortex. Together these findings suggest Panx1 act as a “brake” on the development of dendritic spines with important implications for the development of nerve cell connections.

## Introduction

Pannexin 1 (Panx1) forms channels permeable to ions and metabolites (for review, see Boyce et al., 2018), with modes of activation, channel properties and selectivity currently the subject of intense debate and investigation (Chiu et al., 2018). Nonetheless, Panx1 is enriched in the nervous system, including in neuronal dendrites and spines (Zappalà et al., 2006; Zoidl et al., 2007; Weilinger et al., 2012, 2016; Cone et al., 2013). Panx1 KO is associated with changes in hippocampal synaptic plasticity (Prochnow et al., 2012; Ardiles et al., 2014; Gajardo et al., 2018).

Several lines of evidence suggest that Panx1 could regulate the formation of neuronal networks or network ensembles, which are groups of spontaneously coactive neurons. Ensembles are emerging as the functional building blocks of cortical activity that underlie sensorimotor integration and learning and memory (for review see Harris et al., 2003; Miller et al., 2014; Carrillo-Reid et al., 2015; Arce-McShane et al., 2016). The formation of synapses plays a major role in the development of network ensembles, providing the structural basis for higher network connectivity (Jung and Herms, 2014; for review, see Hoshiba et al., 2017; Frank et al., 2018). In the rodent cortex, Panx1 transcript levels peak around the time of birth, and then markedly decline during the first four postnatal weeks (Ray et al., 2005; Vogt et al., 2005). This decrease in Panx1 coincides with the critical period for the formation of microscopic protrusions emanating from glutamatergic pyramidal neurons called *dendritic spines* (Schlaggar et al., 1993; for review, see O’Leary et al., 1994; Grutzendler et al., 2002; Trachtenberg et al., 2002; Hensch, 2004; Holtmaat et al., 2005), which receive the majority of excitatory inputs in the brain (for review, see Nimchinsky et al., 2002; Alvarez and Sabatini, 2007; Yuste, 2011). Panx1 regulates neurite growth (Wicki-Stordeur and Swayne, 2013) and interacts with collapsin-response mediator protein 2 (Crmp2; Wicki-Stordeur, 2015; Xu et al., 2018), a stable synaptic protein (Heo et al., 2018) that regulates spine development (Zhang et al., 2016).

To understand how Panx1 regulates cortical neuron development, we used a multilevel approach involving analyses of network ensembles, synaptic protein expression and dendritic spines in mice with global and glutamatergic-neuron specific Panx1 KO. Panx1 KO cortical cultures showed increased network ensemble formation. Moreover, Panx1 KO cortical synaptosomes exhibited significantly increased expression of excitatory synapse markers (PSD-95, GluA1, and GluN2A) and significantly increased cortical neuron dendritic spine densities. Together our results suggest that Panx1 regulates network ensemble formation by acting as a brake for dendritic spine formation.

## Materials and Methods

### Antibodies

Primary antibodies used in this study were as follows: mouse anti-Gad67 (1:120; MAB5406, Millipore-Sigma), mouse anti-PSD-95 (1:50 for ICC; 1:1500 for WB; MA1-045, ThermoFisher Scientific), rat anti-glial fibrillary acidic protein (GFAP; 1:200; 1:2000; 13-0300, ThermoFisher Scientific), rabbit anti-MAP2 (1:400; ab32454, Abcam), rabbit anti-Panx1 (1:2000 for WB; 91137, Cell Signaling Technology), rabbit anti-GluA1 (1:2000; 13185, Cell Signaling Technology), rabbit anti-GluA2 (1:2000; 13604, Cell Signaling Technology), rabbit anti-GluN1 (1:1000; 5704, Cell Signaling Technology), rabbit anti-GluN2A (1:1000; ab169873, Abcam), rabbit anti-GluN2B (1:1000; 4207, Cell Signaling Technology). Secondary antibodies used in this study were as follows: AlexaFluor 488-conjugated AffiniPure donkey anti-rabbit IgG (1:600; 711-545-152), AlexaFluor 594-conjugated AffiniPure donkey anti-mouse IgG (1:600; 715-585-150), AlexaFluor 647-conjugated AffiniPure donkey anti-mouse IgG (1:600; 715-605-150), horseradish peroxidase (HRP)-conjugated AffiniPure donkey anti-rabbit IgG (1:4000–1:8000; 711-035-152), HRP-conjugated AffiniPure donkey anti-mouse IgG (1:4000–1:8000; 715-035-150), HRP-conjugated AffiniPure donkey anti-rat (1:4000 – 1:8000; 712-035-150). All secondary antibodies were obtained from Jackson ImmunoResearch.

### Experimental animals

All animal procedures were performed in accordance with the guidelines set by the Canadian Council on Animal Care and approved by the University of Victoria Animal Care Committee. Male and female mice from postnatal day (P)0 to P30 (note that P29 and P30 mice were both labeled as P29) were used in this study. Global Panx1 KO and Panx1^f/f^ strains were derived from a strain originally generated by Dr. Valery Shestopalov (Dvoriantchikova et al., 2012) and now also available from The Jackson Laboratory (026021). Note that the original Panx1^f/f^ 129 strain carried a caspase 4 deletion (Vanden Berghe et al., 2015). These mice have been back-crossed in-house onto C57BL/6J at least 6 times. Wild-type (WT), Panx1 KO, Panx1^f/f^, and Emx1-Cre;Panx1^f/f^ (cKO^E^) are on a C57BL/6J background (000664, The Jackson Laboratory). Panx1 KO mice used for dendritic spine analysis were generated from Panx1^+/−^ breeding pairs (to obtain WT and KO littermates). For conditional KO experiments, breeding pairs consisting of a Panx1^f/f^ male and a Emx1-Cre;Panx1^f/f^ female were used to generate Panx1^f/f^ and Emx1^IRES-Cre^;Panx1^f/f^ littermates. The Emx1^IRES-Cre^ strain was obtained from The Jackson Laboratory (005628). Mice were housed under a 12 h light/dark cycle starting at 8:00 A.M., with food and water *ad libitum*; temperature was maintained between 20 and 25°C and humidity at 40–65%. All animals were weaned at P21 and housed in an enriched environment consisting of crinkle paper, nestlets, one paper hut, and one mouse igloo or mouse tunnel.

### Genotyping

Primers for LoxTGF, LoxTGR, and Panx1 LoxR (CTTTGGCATTTTCCCAGTGT, CGCGGTTGTAGACTTTGTCA, and GTCCCTACAGGAGGCACTGA) were used to genotype mice. Identification of mice carrying the Emx1^IRES-Cre^ transgene was determined using the primers Emx1WTF, Emx1WTR, Generic-CreF, and Generic-CreR (AAGGTGTGGTTCCAG AATCG, CTCTCCACCAGAAGGCTGAG, GCGGTCTGGCAGTAAAAACTATC, GTGAAA CAGCATTGCTGTCACTT). Genomic DNA was extracted from tail clips or ear notches using MyTaq Extract-PCR Kit (BIO-21126, Bioline). PCR of DNA from homozygous WT mice amplifies a single 585 bp band, whereas PCR of DNA from homozygous mutant mice have a single 900 bp band, with both bands apparent in PCR samples run using DNA from heterozygous mice; PCR of DNA from Panx1^f/f^ mice have a single 1898 bp band (Dvoriantchikova et al., 2012). PCR of DNA from mice carrying a single copy of Emx1^IRES-Cre^ transgene have both a 378 bp band (WT) and a 102 bp band (Cre), whereas PCR of DNA from those not carrying the Emx1^IRES-Cre^ transgene have a single 378 bp band.

### Tissue processing and diolistic labeling

Experiments were performed similar to previously described (Brusco et al., 2010; Staffend and Meisel, 2011). Mice were perfused transcardially with 0.1 m PBS followed by 1.5% paraformaldehyde (PFA) in 0.1 m PBS for 30–60 s. The dissected brains were immersed in 1.5% paraformaldehyde for 60 min and then transferred to 0.1 m PBS. DiI (1,1′-Dioctadecyl-3,3,3′,3′-tetramethylindocarbocyanine perchlorate; 42364 Millipore-Sigma) crystals were placed on the dorsolateral surface of the brains and incubated overnight at 37°C in 1.5% PFA. The tissue was fixed with 4% PFA for 30 min at room temperature (RT), followed by three washes with 0.1 m PBS and coronally-sectioned on a vibratome (150 µm). Hoechst 33342 (1:500 in 0.1 m PBS, ThermoFisher Scientific) was used as a nuclear counterstain.

### Dendritic spine analysis in brain sections

Note that imaging and analysis were performed blind to the genotype of the groups. High-resolution 1498 × 1498 image stacks (75.94 nm/pixel; 0.5 µm *z*-steps) were captured using a Leica SP8 confocal microscope with 561 nm laser illumination and a 40×/1.30 NA oil objective and 2.6× digital zoom. The laser power and gain were manually adjusted to prevent oversaturation of pixel intensity values in the apical dendrite. The analysis was conducted with NIH ImageJ v148 (Schneider et al., 2012, https://imagej.nih.gov/ij/) and was restricted to primary apical dendrites on their trajectory through Layers 2/3. Apical shafts were selected for analysis according to the following criteria: (1) The diolistic label reached the soma of a layer 5 pyramidal cell, (2) the shaft measured 2–4 µm wide, and (3) at least 100 µm of the shaft was clearly discernible from surrounding cells/shafts. Spines were manually traced through *z*-sections from the head to their origin on the shaft, considering the following: (1) they protruded from the shaft by at least 0.4 µm, and (2) they were separated by at least 4 µm from a neighboring apical dendrite. The spines of six apical dendrites that matched these criteria were analyzed for each animal. The spine density was defined as the number of spines per 10 µm and was calculated by multiplying the total number of spines by the length of the apical dendrite in µm multiplied by 10. Representative images were processed uniformly with a Gaussian blur of 0.5 pixels, and uniform adjustments to levels and contrast were made using Photoshop CS6 Extended suite (Adobe Systems).

### Primary cortical neuron cultures

Cortices from P0 WT and Panx1 KO pups of either sex were microdissected, chopped with a razor blade and incubated with papain (150 µg/L; P4762, Millipore-Sigma), dispase I (150 µg/L; D4818, Millipore-Sigma), and DNase 1 (100 µg/L; 10104159001, Roche) for 40 min, followed by mechanical dissociation in DMEM/F12 medium supplemented with Neurocult SM1 supplement (05711, STEMCELL Technologies), and l-glutamine (200 mm; 07100, STEMCELL Technologies), and penicillin/streptomycin (P/S, 0.1 U/ml; 15140122, Invitrogen). Cells were plated at a density of 2.5 × 10^5^ cells per cm^2^ on poly-d-lysine (PDL) pre-coated glass coverslips (GG-12-1.5-PDL, NeuVitro) or Nunc LabTek Chamber Slide systems (154534PK, ThermoFisher Scientific) coated with PDL (P6407, Millipore-Sigma) for MTT assays. The medium was replaced with Neurocult medium (STEMCELL Technologies, 05700) supplemented with SM1 and l-glutamine, P/S, and gentamicin (0.1 mg/ml; G1397, Millipore-Sigma). From 4 days *in vitro* onward, partial (half) the medium was replenished with new BrainPhys maturation medium (Bardy et al., 2015) supplemented with SM1 and Cytosine β-d-arabinofuranoside (ara-C, C1768, Millipore-Sigma) every third day.

### Immunostaining and spiny protrusion analysis in cultured neurons

Primary cortical neurons were fixed in 4% EM-grade paraformaldehyde solution pre-warmed to 37°C for 10 min, washed in PBS and permeabilization with 0.25% Triton X-100 in PBS (PBST) for 10 min at RT, washed again with PBS and then blocked with 10% donkey serum (DS; 017-000-121, Jackson ImmunoResearch), 1% BSA, and 22.52 mg/ml glycine in PBST for 30 min at RT. Following blocking, cultures were incubated with primary antibodies in 1% BSA, and 5% DS in PBST overnight at 4°C, washed in PBS three times (10 min each), and incubated with secondary antibodies and Alexa Fluor 555 phalloidin (A34055, Invitrogen) in PBST supplemented with 1% BSA, and 5% DS for 1 h at RT. After three washes (10 min), coverslips were mounted on microscope slides using VECTASHIELD Antifade Mounting Medium (H-1000, Vector Laboratories). Hoechst 33342 (H3570, Invitrogen) was used as nuclear stain. For the analysis of spiny protrusions and PSD-95-positive dendritic spines, high-resolution (2048 × 2048, pixel size 0.090 µm) images of neurons were captured using a Leica SP8 confocal microscope (63×/1.20 NA). The same acquisition parameters were maintained for all cells across all separate cultures within an experiment. Dendritic spines were defined as actin-enriched protrusions ranging from 0.4 to 10 µm in length that emanated directly from the dendritic shaft. Using ImageJ, the longest dendrite of each cell was selected and defined as the primary neurite. Within the primary neurite, a 20 µm segment from the distal tip of the primary neurite was traced and dendritic spines within the segment were traced with individual regions-of-interest (ROIs); spine density was defined as the number of spines per 10 µm and was calculated by multiplying the total number of spines traced by 0.5. For cell-type characterization of neuronal cultures, coverslips were stained with the protocol described above and primary antibodies (MAP2, Gad67, and GFAP) were incubated overnight at 4°C, followed by three 10 min washes in PBS, secondary antibody and Hoechst incubation at room temperature, and three more 10 min washes before mounting the coverslips with VECTASHIELD. Images (1024 × 1024, pixel size 0.568 µm, 0.34 mm^2^) were captured with a Leica SP8 confocal microscope (20×/0.7 NA). The proportion of astrocytes and inhibitory cells were calculated based on GFAP and Gad67 immunoreactivity relative to the total amount of cells (MAP2-positive cells + GFAP-positive cells). The proportion of excitatory cells was determined from MAP2-positive/Gad67-negative relative to the total amount of cells. Representative images were uniformly adjusted with Gaussian blur (2 pixels), and mild uniform adjustments to levels and contrast were made using Photoshop CS6 Extended suite (Adobe Systems).

### Neuronal network analysis in primary cortical neuron cultures

For Ca^2+^ imaging experiments, neuronal cultures 12–14 days *in vitro* were washed with HBSS and incubated in in BrainPhys maturation medium supplemented 4 µm Fluo-4 AM (F14201, ThermoFisher Scientific) for 40 min at 37°C, 5% CO_2_, and 95% humidity. Coverslips were washed, transferred to a 35 mm dish containing BrainPhys without phenol red (05791, STEMCELL Technologies), and incubated in the dark for 30 min at 37°C, 5% CO_2_, and 95% humidity to allow for complete de-esterification of the probe. The dish was then mounted onto a heated chamber held at 37°C, 5% CO_2_ and images were acquired every 5 s for 10 min (pixel dwell time 36 ns, streamed at 7.41 Hz, exposure/frame capture time 135 ms, 120 frames) with a laser-scanning microscope (Leica SP8) using 471 nm laser illumination (constant 5% laser power) and a 20× objective (NA 0.70). Three fields-of-view (FOVs, 1024 × 1024, pixel size 0.455 µm) were analyzed per coverslip. ROIs were drawn around each soma within each FOV. The raw fluorescence intensity values over time within each ROI were extracted using the Leica Application Suite Software v3.1.3.16308 (Leica Microsystems); the background signal was determined in areas lacking neurons and was subtracted from all the intensity records; fluorescence intensity values were exported as .csv files. Two to three coverslips across three independent cultures were used for this analysis (WT = 8 coverslips across 3 independent cultures; KO = 7 coverslips across 3 independent cultures). Note that cells exhibiting a range in fluorescence intensity values limited to within 10% of maximal fluorescence intensity across the entire recording (i.e. fluorescence intensity of 90% of maximum or greater) were removed from all subsequent analyses, resulting in a total of 27/1044 cells removed across WT coverslips (2.9%) and 66/1155 cells removed across Panx1 KO coverslips (5.7%). This exclusion criteria was used to remove cells with abnormally high fluorescence values that could confound our analysis; however, note that it might potentially also eliminate cells with very small calcium transients, skewing our results toward more active cells. Then, the extracted .csv files were processed using the Fluorescence Single Neuron and Network Analysis Package, FluoroSNNAP (https://www.seas.upenn.edu/~molneuro/software.html; University of Pennsylvania), an open-source, interactive plugin for MATLAB (MATLAB R2014a, the MathWorks) for Δ*F/F*_0_ conversion from raw *F* data, spike probability inference, and network ensemble analysis. Once the raw fluorescence .csv file was imported, the analysis package generated a mock image or stack by randomly placing all the traced ROIs contained in the .csv file, which served to interact with the imported data by selecting individual ROIs and visualizing their time-varying traces. By selecting the option “Convert raw fluorescence data to deltaF/F” the difference in fluorescence (Δ*F/F*_0_) was computed by taking the average of all the pixels within each ROI (raw fluorescence trace) and subtracting each value with the mean of the <50% values in the previous 10 frames (adjustable parameter), and then dividing that product by the mean of the lower 50% values in the previous 10 frames (Patel et al., 2015). Selection of the module “Infer underlying spike probability” calculated the spike probability of each individual ROI using a fast, non-negative deconvolution method developed by Vogelstein et al. (2010). This inferred spike probability algorithm represents neuronal activity better than Δ*F/F* (Vogelstein et al., 2010; Miller et al., 2014). Network ensembles, defined as the group of co-activate neurons in a high-activity frame, were calculated by thresholding spike probability data to 3 SD above zero, determined from spike probabilities of the entire population in each FOV; this serves to identify active cells not confounded by noise. Values above the threshold were set to 1, and those below the threshold were set to 0. Then, these binary activity data were shuffled 1000 times to identify the statistically significant number of groups of coactive neurons, using a significant level of *p* < 0.05 (Miller et al., 2014). For distribution analyses the median raw Fluo-4 AM fluorescence intensity (in this case defined as baseline fluorescence) and the difference between maximum and minimum fluorescence intensity values (in this case defined as Δ*F*, fluorescence intensity range) across all frames were obtained for each ROI (cell) collected in the present study and plotted as relative frequency distributions. Violin plots were generated in RStudio, and distributions were compared using the nonparametric Mann–Whitney *U* test.

### MTT cell viability assay

Cell viability was evaluated in WT and Panx1 KO neuronal cultures Nunc^TM^ LabTek^TM^ Chamber Slide^TM^ using the Vybrant^®^ MTT ([3-(4,5-dimethylthiazol-2-yl)-2,5-diphenyltetrazolium bromide]) cell proliferation Assay Kit (V13154, ThermoFisher Scientific) following the manufacturer’s instructions. Briefly, 12 mm of MTT stock solution (Component A) were prepared by adding 1 ml of PBS to a 5 mg vial of MTT; 20 µL of this 12 mm MTT solution was added to each well containing neurons bathed in 180 µL of fresh BrainPhys without Phenol Red (05791, STEMCELL Technologies) and incubated for 4 h at 37°C and 5% CO_2_; wells without neurons were used as negative controls. After this step, ¾ of the medium were removed and 100 µl of DMSO were added in, mixing thoroughly the contents of the well and incubating for 10 min at 37°C. Next, the resulting solution was mixed once again, and the absorbance was read at 540 nm using a microplate reader (Infinite PRO microplate reader, Tecan Life Sciences). All absorbance values represent the average of nine scans per well and were normalized to blank wells (wells without neurons). Six wells per culture per group were used for this assay (*n* = 3 per group).

### Synaptosome preparation and Western blotting

Synaptic proteins were extracted using Syn-PER Synaptic Protein Extraction Reagent (87793, ThermoFisher Scientific) according to the manufacturer’s instructions. Briefly, WT and Panx1 KO P14 and P29 cortices were dissected and weighed and then submerged in ice-cold Syn-PER reagent (1 mL/100 mg) supplemented with protease inhibitor cocktail (P8340, Millipore-Sigma). After homogenization on ice, 10–20% of the homogenate was stored at −80°C; the remaining of the homogenate was centrifuged at 1200 × *g* for 10 min at 4°C. The pellet was discarded, and the supernatant transferred to a new tube, for a new round of centrifugation at 15,000 × *g* for 20 min at 4°C, obtaining synaptosomes. This pellet was resuspended in Syn-PER reagent using 150 μL per 100 mg of brain tissue. This synaptosome suspension was stored in 5% (v/v) DMSO at −80°C until analysis. On the day of analysis, 50 μL of the synaptosome suspension was placed in a new tube and centrifuged to collect the pellet. Protein was extracted by adding 200 μL of PBS-based RIPA lysis buffer (1% IGEPAL, 0.5% sodium deoxycholate, 0.1% SDS, supplemented with PI cocktail, PMSF and Na orthovanadate) followed by incubation on ice for 30 min. Samples were heated to 95–100°C for 10 min in Laemmli sample buffer, DTT and β−ME before loading 10 µg of protein per lane onto 10% PAGE gels (TGX Stain-Free FastCast Acrylamide Kit 161-0183, Bio-Rad) and protein separation was achieved by application of 200 V. Following electrophoresis, gels were exposed to 30 s UV light (G-box imager) to obtain the TGX Stain-Free signal (total protein) and then transferred to polyvinylidene fluoride (PVDF) for 1 h at 100 V. Following this, the TGX Stain-Free signal was captured by UV light (5 s), rinsed with deionized water for 30 s, blocked in 5% skim milk in PBS supplemented with 0.1% Tween 20, incubated with primary antibodies at 4°C overnight, and secondary antibodies for 1 h at RT after three washes in PBST. The immunoreactive bands were visualized by enhanced chemiluminescence and quantified using ImageJ (http://imagej.nih.gov/ij/).

### Experimental design and statistical analysis

For *ex vivo* analysis (diolistic labeling of dendritic spines) WT and Panx1 KO groups consisted of equal numbers of male and female mice. Note that separate analyses of male and female groups revealed no sex-specific differences in the overall effects and as such the sexes were combined. For *in vitro* experiments, appropriate controls are clearly identified in detail in the figures and figure legends. Treatment timelines and all other relevant details are described in Results and in the figure legends and where appropriate, illustrated on the figures themselves. Researchers were blinded to the identity of the treatment/experimental groups at all stages of the analysis, except for Western blot analysis. Data are presented as mean ± SEM. Significance comparisons were calculated using unpaired Student’s *t* test, one-way ANOVA and two-way ANOVA for grouped analyses. Bonferroni’s correction was used for multiple comparisons when appropriate. When interactions were statistically significant while using two-way ANOVA for grouped analysis, simple effect ANOVAs with multiple comparison were performed using Bonferroni’s correction. For non-normally distributed data, we used nonparametric tests. Details of normality tests can be found in Table 1. Statistical significance was determined by *p* < 0.05 in all tests used in the present study. Data were analyzed using GraphPad Prism version 6.0d (GraphPad Software), and RStudio v1.1.463 (RStudio). Significance is denoted as *p < 0.05, **p < 0.01, ***p < 0.001, ****p < 0.0001. Results of statistical tests are described in detail in the Table 1; superscript letters throughout the results section and figure legends indicate the corresponding statistic in the table.

**Table 1.** Statistical table

	Figure	Comparison	Data Structure (Shapiro–Wilk normality test unless otherwise stated)	Type of test	Statistic	Confidence, 95% CI
a1	1*Bi*	WT vs Panx1 KO	Normal distribution	Unpaired two-tailed *t* test	*t* = 4.051; df = 13	*p* = 0.0014; 1.667 to 5.476
a2	1*Bi*	WT vs Panx1 KO	Normal distribution (D’Agostino-Pearson Normality Test chosen because of multiple identical values)	Unpaired two-tailed *t* test	*i* = 4.374; df = 39	*p* < 0.0001; 1.894 to 5.153
b1	1*Ci*	WT vs Panx1 KO	Normal distribution (D’Agostino-Pearson Normality Test chosen because of multiple identical values)	Unpaired two-tailed *t* test	*t* = 2.844; df = 39	*p* = 0.0071; 0.4654 to 2.758
b2	1*Ci*	WT vs Panx1 KO	Normal distribution	Unpaired two-tailed *t* test	*t* = 1.320; df = 30	*p* = 0.1968; −0.3101 to 1.443
c	1*D*	WT vs Panx1 KO	Not normal (*p* < 0.0001)	Mann-Whitney *U* test (two-tailed)	*U* = 316,969	*p* < 0.0001, 834622, 1.384e6
d	1*E*	WT vs Panx1 KO	Not normal (*p* < 0.0001)	Mann-Whitney *U* test (two-tailed)	*U* = 294,294	*p* < 0.0001; 811947, 1.407e6
e1	1*Fii*	WT vs Panx1 KO, interaction effect	Normal distribution	Two-way ANOVA	*F*_(2, 90)_ = 3.475	*p* = 0.0352
e2	1*Fii*	WT vs Panx1 KO, cell-type effect	Normal distribution	Two-way ANOVA	*F*_(2, 90)_ = 2615	*p <* 0.0001
e3	1*Fii*	WT vs Panx1 KO, genotype effect	Normal distribution	Two-way ANOVA	*F*_(1, 90)_ = 4.934e-008	*p* = 0.9998
e4	1*Fii*	WT vs Panx1 KO, excitatory neurons	Normal distribution	Two-way ANOVA with Bonferroni’s correction		*p* = 0.9702, −2.347 to 5.568
e5	1*Fii*	WT vs Panx1 KO, inhibitory neurons	Normal distribution	Two-way ANOVA with Bonferroni’s correction		*p* = 0.7500, −2.079 to 5.835
e6	1*Fii*	WT vs Panx1 KO, astrocytes	Normal distribution	Two-way ANOVA with Bonferroni’s correction		*p* = 0.1026, −7.445 to 0.4690
e7	1*Fii*	WT vs Panx1 KO	Normal distribution	Simple effect ANOVA^a^	*F*_(5, 90)_ = 1047	*p* < 0.0001
e8	1*Fii*	WT vs Panx1 KO, excitatory neurons	Normal distribution	Simple effect ANOVA^a^ with Bonferroni’s correction		*p* = 0.9702, −2.347 to 5.568
e9	1*Fii*	WT vs Panx1 KO, inhibitory neurons	Normal distribution	Simple effect ANOVA^a^ with Bonferroni’s correction		*p* = 0.7500, −2.079 to 5.835
e10	1*Fii*	WT vs Panx1 KO, astrocytes	Normal distribution	Simple effect ANOVA^a^ with Bonferroni’s correction		*p* = 0.1026, −7.445 to 0.4690
f	1*G*	WT vs Panx1 KO Formazan absorbance (MTT conversion to formazan)	Normal distribution	Unpaired two-tailed *t* test	*t* = 0.128 df = 4	*p* = 0.9089, −25.76 to 23.59
g1	2*Aiii*	PSD-95 and Panx1 expression in Homogenate (H) vs Synaptosome (P3) content interaction	Normal distribution	Two-way ANOVA	*F*_(1, 8)_ = 9.847	*p* = 0.0138
g2	2*Aiii*	PSD-95 and Panx1 expression effect	Normal distribution	Two-way ANOVA	*F*_(1, 8)_ = 9.847	*p* = 0.0138
g3	2*Aiii*	H vs P3 content effect	Normal distribution	Two-way ANOVA	*F*_(1, 8)_ = 74.46	*p* < 0.0001
g4	2*Aiii*	PSD-95 expression in H vs P3	Normal distribution	Two-way ANOVA with Bonferroni’s correction		*p* < 0.0001; −358.0 to −180.1
g5	2*Aiii*	Panx1 expression in H vs P3	Normal distribution	Two-way ANOVA with Bonferroni’s correction		*p* = 0.0093; −214.5 to −36.58
g6	2*Aiii*	PSD-95 and Panx1 expression in H vs P3	Normal distribution	Simple effect ANOVA^a^	*F*_(3, 8)_ = 31.38	*p* < 0.0001
g7	2*Aiii*	Panx1 expression in H vs P3	Normal distribution	Simple effect ANOVA^a^ with Bonferroni’s correction		*p* < 0.0001; −214.5 to −36.58
g8	2*Aiii*	PSD-95 and Panx1 expression in H and P3	Normal distribution	Simple effect ANOVA^a^ with Bonferroni’s correction		*p* = 0.0093; −358.0 to −180.1
h1	2*Biii*	Panx1 expression P7–P63	Normal distribution	One-way ANOVA	*F*_(3, 8)_ = 365.9	*p* < 0.0001
h2	2*Biii*	Panx1 expression P7–P14	Normal distribution	One-way ANOVA with Bonferroni’s correction		*p* < 0.0001; 0.6377 to 0.8563
h3	2*Biii*	Panx1 expression P14-P29	Normal distribution	One-way ANOVA with Bonferroni’s correction		*p* = 0.0006; 0.1161 to 0.3218
h4	2*Biii*	Panx1 expression P29–P63	Normal distribution	One-way ANOVA with Bonferroni’s correction		*p* = 0.9604; −0.08815 to 0.1304
i1	3*B*	Panx1 expression WT vs KO (genotype) by age interaction	Normal distribution	Two-way ANOVA	*F*_(1, 16)_ = 84.46	*p* < 0.0001
i2	3*B*	Genotype effect	Normal distribution	Two-way ANOVA	*F*_(1, 16)_ = 144.7	*p* < 0.0001
i3	3*B*	Age effect	Normal distribution	Two-way ANOVA	*F*_(1, 16)_ = 84.46	*p* < 0.0001
i4	3*B*	Panx1 expression WT P14 vs WT P29	Normal distribution	Two-way ANOVA with Bonferroni’s correction		*p* < 0.0001;70.14 to 103.1
i5	3*B*	Panx1 expression KO P14 vs KO P29	Normal distribution	Two-way ANOVA with Bonferroni’s correction		*p* = > 0.9999; −16.48 to 16.48
i6	3*B*	Panx1 expression WT P14–P29 and KO P14–P29	Normal distribution	Simple effect ANOVA^a^	*F*_(3, 16)_ = 104.5	*p* < 0.0001
i7	3*B*	Panx1 expression WT P14 vs WT P29	Normal distribution	Simple effect ANOVA^a^ with Bonferroni’s correction		*p* < 0.0001, 67.87 to 105.4
i8	3*B*	Panx1 expression WT P14 % KO 14	Normal distribution	Simple effect ANOVA^a^ with Bonferroni’s correction		*p* < 0.0001; 81.25 to 118.7
i9	3*B*	Panx1 expression WT 29 vs KO P29	Normal distribution	Simple effect ANOVA^a^ with Bonferroni’s correction		*p* = 0.2476; −5.369 to 32.13
i10	3*B*	Panx1 expression KO P14 vs KO P29	Normal distribution	Simple effect ANOVA^a^ with Bonferroni’s correction		*p* > 0.9999; −18.75 to 18.75
j1	3*B*	PSD-95 expression WT vs KO (genotype) by age interaction	Normal distribution	Two-way ANOVA	*F*_(1, 16)_ = 4.208	*p* = 0.0570
j2	3*B*	Genotype effect	Normal distribution	Two-way ANOVA	*F*_(1, 16)_ = 37.42	*p* < 0.0001
j3	3*B*	Age effect	Normal distribution	Two-way ANOVA	*F*_(1, 16)_ = 175.8	*p* < 0.0001
j4	3*B*	PSD-95, WT P14 vs KO P14	Normal distribution	Two-way ANOVA with Bonferroni’s correction		*p* < 0.0001; −113.1 to −45.30
j5	3*B*	PSD-95, WT P29 vs KO P29	Normal distribution	Two-way ANOVA with Bonferroni’s correction		*p* = 0.0220; −73.34 to −5.516
k1	3*B*	GluA1 expression WT vs KO (genotype) by age interaction	Normal distribution	Two-way ANOVA	*F*_(1, 16)_ = 0.1996	*p* = 0.6611
k2	3*B*	Genotype effect	Normal distribution	Two-way ANOVA	*F*_(1, 16)_ = 9.090	*p* = 0.0082
k3	3*B*	Age effect	Normal distribution	Two-way ANOVA	*F*_(1, 16)_ = 0.02040	*p* = 0.8882
k4	3*B*	GluA1, WT P14 vs KO P14	Normal distribution	Two-way ANOVA with Bonferroni’s correction		*p* = 0.1763; −131.3 to 20.11
k5	3*B*	GluA1, WT P29 vs KO P29	Normal distribution	Two-way ANOVA with Bonferroni’s correction		*p* = 0.0526; −150.6 to 0.7678
l1	3*B*	GluA2 expression WT vs KO (genotype) by age interaction	Normal distribution	Two-way ANOVA	*F*_(1, 16)_ = 1.156	*p* = 0.2982
l2	3*B*	Genotype effect	Normal distribution	Two-way ANOVA	*F*_(1, 16)_ = 0.5621	*p* = 0.4643
l3	3*B*	Age effect	Normal distribution	Two-way ANOVA	*F*_(1, 16)_ = 0.1894	*p* = 0.6693
m1	3*B*	GluN1 expression WT vs KO (genotype) by age interaction	Normal distribution	Two-way ANOVA	*F*_(1, 16)_ = 4.900	*p* = 0.0417
m2	3*B*	Genotype effect	Normal distribution	Two-way ANOVA	*F*_(1, 16)_ = 0.05221	*p* = 0.8222
m3	3*B*	Age effect	Normal distribution	Two-way ANOVA	*F*_(1, 16)_ = 19.95	*p* = 0.0004
m4	3*B*	GluN1, WT P14 vs KO P14	Normal distribution	Two-way ANOVA with Bonferroni’s correction		*p =* 0.3590; −22.63 to 6.241
m5	3*B*	GluN1, WT P29 vs KO P29	Normal distribution	Two-way ANOVA with Bonferroni’s correction		*p* = 0.2069; −4.355 to 24.52
m6	3*B*	GluN1 expression WT P14–P29 and KO P14–P29	Normal distribution	Simple effect ANOVA^a^	*F*_(3, 16)_ = 8.300	*p* = 0.0015
m7	3*B*	GluN1 expression WT P14–P29	Normal distribution	Simple effect ANOVA^a^ with Bonferroni’s correction		*p* = 0.0009; −43.99 to −11.15
m8	3*B*	GluN1 expression KO P14–P29	Normal distribution	Simple effect ANOVA^a^ with Bonferroni’s correction		*p* = 0.5231; −25.72 to 7.123
m9	3*B*	GluN1 expression WT vs KO, P14	Normal distribution	Simple effect ANOVA^a^ with Bonferroni’s correction		*p* = 0.7180, −24.62 to 8.227
m10	3*B*	GluN1 expression WT vs KO, P29	Normal distribution	Simple effect ANOVA^a^ with Bonferroni’s correction		*p* = 0.4138, −6.341 to 26.50
n1	3*B*	GluN2A expression WT vs KO (genotype) by age interaction	Normal distribution	Two-way ANOVA	*F*_(1, 16)_ = 0.05302	*p* = 0.8208
n2	3*B*	Genotype effect	Normal distribution	Two-way ANOVA	*F*_(1, 16)_ = 7.892	*p* = 0.0126
n3	3*B*	Age effect	Normal distribution	Two-way ANOVA	*F*_(1, 16)_ = 1.092	*p* = 0.3115
n4	3*B*	GluN2A, WT P14 vs KO P14	Normal distribution	Two-way ANOVA with Bonferroni’s correction		*p* = 0.1739; −159.7 to 24.14
n5	3*B*	GluN2A, WT P29 vs KO P29	Normal distribution	Two-way ANOVA with Bonferroni’s correction		*p* = 0.0945; −171.8 to 12.03
o1	3*B*	GluN2B expression WT vs KO (genotype) by age interaction	Normal distribution	Two-way ANOVA	*F*_(1, 16)_ = 3.507	*p* = 0.0795
o2	3*B*	Genotype effect	Normal distribution	Two-way ANOVA	*F*_(1, 16)_ = 1.219	*p* = 0.2859
o3	3*B*	Age effect	Normal distribution	Two-way ANOVA	*F*_(1, 16)_ = 4.547	*p* = 0.0488
o4	3*B*	GluN2B, WT P14 vs WT P29	Normal distribution	Two-way ANOVA with Bonferroni’s correction		*p* > 0.9999; −35.97 to 41.75
o5	3*B*	GluN2B, KO P14 vs KO P29	Normal distribution	Two-way ANOVA with Bonferroni’s correction		*p =* 0.0240; 5.644 to 83.37
p1	4*Bi*	Spine density WT P14 vs KO P14	Normal distribution	Unpaired two-tailed *t* test	*t* = 3.962; df = 14	*p* = 0.0014; −5.368 to −1.597
p2	4*Bi*	Spine length WT P14 vs KO P14	Normal distribution	Unpaired two-tailed *t* test	*t* = 0.8432; df = 14	*p* = 0.4133; −0.09070 to 0.2082
p3	4*Bi*	Spine head diameter WT P14 vs KO P14, total distribution	Not normal	Mann-Whitney *U* test (two-tailed)	*U* = 1.474e^7^	*p* = 0.0131
p4	4*Bi*	Spine head diameter WT P14 vs KO P14, 25% right tail (> percentile 75)	Not normal	Mann-Whitney *U* test (two-tailed)	*U* = 931,253	*p* = 0.4022.
q1	4*Bi*	Spine density WT P29 vs KO P29	Normal distribution	Unpaired two-tailed *t* test	*t* = 5.754; df = 12	*p* < 0.0001; 3.279 to 7.275
q2	4*Biii*	Spine lengthWT P29 vs KO P29	Normal distribution	Unpaired two-tailed *t* test	*t* = 0.8214; df = 12	*p* = 0.4274; −0.05194 to 0.1148
r1	4*Biii*	Spine densityPanx1^f/f^ vs Panx1 cKO^E^	Normal distribution	Unpaired two-tailed *t* test	*t* = 4.548; df = 4	*p* = 0.0104; 2.767 to 11.44
r2	4*Cii*	Spine length Panx1^f/f^ vs Panx1 cKO^E^	Normal distribution	Unpaired two-tailed *t* test	*t* = 0.8717; df = 4	*p* = 0.4326; −0.1602 to 0.3069
s1	4*Cii*	Spine density WT vs KO primary cortical neurons	Normal distribution (D’Agostino-Pearson Normality Test chosen due to multiple identical values)	Unpaired two-tailed *t* test	*t* = 8.336; df = 25	*p* < 0.0001;4.482 to 7.424
s2	4*Cii*	PSD-95+ spines WT vs KO primary cortical neurons	Normal distribution (D’Agostino-Pearson Normality Test chosen due to multiple identical values)	Unpaired two-tailed *t* test	*t* = 4.243; df = 25	*p* = 0.0003; 1.220 to 3.521
s3	4*Cii*	Spine Length WT vs KO primary cortical neurons	Normal distribution	Unpaired two-tailed *t* test	*t* = 1.302; df = 25	*p* = 0.2047; −0.4186 to 0.09428

a Group analyses were performed using two-way ANOVAs. When interactions were significant, a one-way ANOVA with Bonferroni’s multiple-comparison’s test correction was performed to evaluate simple effects (McDonald, 2014).

## Results

### Increased network ensembles and altered Ca^2+^ dynamics in Panx1 KO cortical neurons

**Figure 1.**
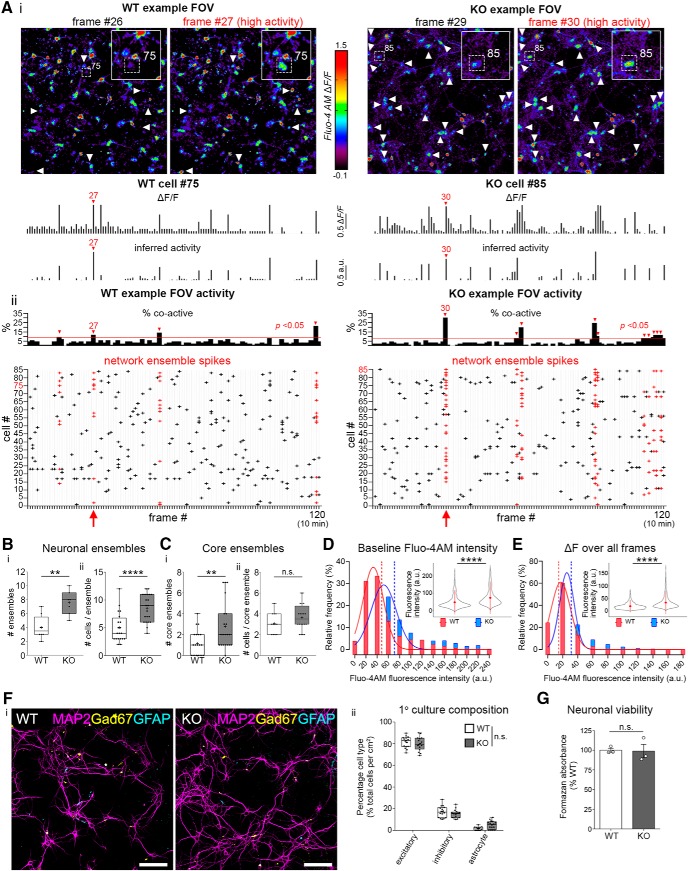
Increased network ensembles and altered Ca^2+^ dynamics in Panx1 KO cortical neurons. ***A***, Representative analyses for functional connectivity in WT and Panx1 KO primary cortical neuron cultures. Ca^2+^ imaging data were collected using confocal microscopy in DIV12-14 primary cortical neurons using Fluo-4-AM. A MATLAB based program called FluoroSNNAP was used to determine network ensemble properties. ***Ai***, Confocal micrographs of exemplary FOVs of WT and Panx1 KO (labeled KO) demonstrating Fluo-4-derived Ca^2+^ activity from low and high activity frames (as indicated), along with the FluoroSNNAP output Δ*F/F* (middle) *and* inferred spikes (bottom) from the identified WT (75) and KO (85) cells. ***Aii***, Percentage of active neurons in each frame from the example FOVs (top). The red line indicates the threshold for a statistically significant number of coactive cells in a frame used by FluoroSNNAP (3 SD). Raster plots of WT and KO example FOVs (bottom) generated from thresholded spike probability data. Spikes from cells participating in a network ensemble are shown in red. The exemplary high activity frames and cells from ***A*** are also highlighted in red. ***B***, Network ensemble data from WT and Panx1 KO DIV12-14 primary neuron cultures. ***Bi***, The mean number of network ensembles was increased in Panx1 KO cultures (WT: 4.0 ± 0.6, KO: 7.6 ± 0.7 network ensembles; *t*_(13)_ = 4.1, *p* = 0.0014^a1^; *n* = 7–8 coverslips from 3 independent cultures; **p < 0.01). ***Bii***, The number of cells involved in network ensembles was also increased in Panx1 KO neurons (WT: 5.0 ± 0.6, KO: 8.5 ± 0.6 cell per ensemble; *t*_(13)_ = 4.4, *p* < 0.0001^a2^; *n* = 20–21 network ensembles from 3 independent cultures; ****p < 0.0001). ***C***, Core network ensemble data from WT and Panx1 KO DIV12-14 primary neuron cultures. ***Ci***, The mean number of core ensembles (co-activated neurons participating in more than one ensemble) was increased in Panx1 KO cultures (WT: 1.2 ± 0.3, KO: 2.7 ± 0.5 core ensembles; *t*_(39)_ = 2.8, *p* = 0.0071^b1^; *n* = 20–21 network ensembles from 3 independent cultures; **p < 0.01). ***Cii***, The number of cells forming a core ensemble was not significant different between the analyzed groups (WT: 3.1 ± 0.3, KO: 3.7 ± 0.3 cells per core ensemble; *t*_(30)_ = 1.3, *p* = 0.1968^b2^; *n* = 12–20 core ensembles from 3 independent cultures; n.s., not significant). ***D***, Distributions and violin plots of resting and total change (maximum minus minimum) of Fluo-4 fluorescence intensities in DIV12-14 primary cortical neuronal cultures. ***Di***, Frequency distributions of Fluo-4 Ca^2+^ indicator dye fluorescence intensities of WT (red) and Panx1 KO (blue) revealed a right shift toward higher median Ca^2+^ levels at baseline (defined the as raw median fluorescence intensity value for each neuron; WT media*n* = 37, *n* = 1017 cells; KO media*n* = 58.50, *n* = 1089 cells; *p* < 0.0001^c^; Mann–Whitney *U* = 316,969; data compiled from 7 to 8 coverslips from 3 independent cultures per condition; ****p < 0.0001). Dotted lines represent the mean of each distribution; a.u., arbitrary units. ***E***, Similarly, the difference between the maximum and minimum fluorescence intensity values (Δ*F*, fluorescence intensity range) was right-shifted and significant larger in Panx1 KO neurons (WT median = 16, *n* = 1017 cells; KO median = 25, *n* = 1089 cells; *p* < 0.0001^d^; Mann–Whitney *U* = 294,294; data compiled from a total of 7–8 coverslips across 3 independent cultures per condition; ****p < 0.0001). Dotted lines represent the mean of each distribution; a.u., arbitrary units. ***F***, WT and Panx1 KO cortical neuronal cultures have a similar cell-type composition. ***Fi***, Representative images of WT and Panx1 KO cortical neurons labeled with the pan-neuronal marker MAP2, interneuron marker Gad67, and the astrocytic marker GFAP. Scale bar, 100 µm. ***Fii***, The proportion of excitatory neurons, inhibitory neurons, and astrocytes was similar between groups (WT excitatory neurons = 81.4% ± 1.3%, KO excitatory neurons = 79.8% ± 1.6%, *p* = 0.9702^e8^; WT inhibitory neurons = 17.1% ± 1.3%, KO inhibitory neurons = 15.2% ± 1.0%, *p* = 0.7500^e9^; WT astrocytes = 1.5% ± 0.4%, KO astrocytes = 4.9% ± 1.0%, *p* = 0.1026^e10^; simple-effect ANOVA with Bonferroni’s multiple-comparison test, *n* = 16 FOV from 2 independent cultures; n.s., not significant). ***G***, WT and Panx1 cortical neurons exhibited similar cell viability. Conversion of MTT to formazan (absorbance measured at 540 nm) was not significant between groups (WT = 100% ± 2.5%, KO = 98.62% ± 8.5%; *p* = 0.9089^f^; *t*_(4)_ = 0.128; *n* = 3 independent cultures; n.s., not significant). Data are presented as mean ± SEM.

To determine the impact of Panx1 on network connectivity, we performed Fluo-4 am Ca^2+^ imaging in primary cortical neuron cultures from WT and Panx1 KO mice (Fig. 1). Spontaneous developing networks in cultured cortical neurons exhibit self-sustaining bursts lasting a few hundred milliseconds occurring at 0.05– 0.1 Hz between days *in vitro* 8 (DIV8) and DIV21 (Habets et al., 1987; Murphy et al., 1992; Maeda et al., 1995; Tibau et al., 2013). Considering these characteristics and other experimental factors (minimization of photo toxicity, imaging multiple FOVs), we imaged DIV12-14 cultures at 0.2 Hz (pixel dwell time = 36 ns; total frame capture time = 135 ms) for 10 min (120 frames). To tease out the effects of Panx1 KO on network properties we performed computational modeling of our Ca^2+^ imaging data using the MATLAB based open-source package, FluoroSNNAP (Fluorescence Single Neuron and Network Analysis Package). FluoroSNNAP allowed us to determine the number and properties of network ensembles, which are defined as a group of neurons that undergo a statistically significant degree of coactivation neurons. These ensembles were identified by their contribution to a so-called “high-activity frame” characterized by a statistically significant proportion of activated neurons (Miller et al., 2014; Patel et al., 2015). Within this algorithm, statistically significant (*p* < 0.05) high activity frames were identified by comparing the mean activity level of a given frame with a computationally-derived activity threshold calculated using the inferred spike activity data of each cell permutated 1000 times across the entirety of the recording period).

Figure 1*A* depicts Fluo-4 and FluoroSNNAP analyses from exemplary FOV and exemplary cells from WT (left side) and Panx1 KO (right side) cortical neuron cultures. Figure 1*Ai* depicts two sequential Fluo-4 fluorescence frames captured from an exemplary WT FOV (left side) and an exemplary Panx1 KO FOV (right side) cultured cortical neurons. An increase in Fluo-4 fluorescence intensity in exemplary WT Cell 75 highlighted in high-activity Frame 27 (on the right) was evident by comparison with the preceding frame (26). Similarly, an increase in Fluo-4 fluorescence intensity in exemplary Panx1 KO Cell 85 highlighted in high-activity Frame 30 (on the right) was evident by comparison with preceding frame (29). The FluoroSNNAP-computed Δ*F/F* (Fig. *1Ai*, middle) and inferred activity (Fig. 1*Ai*, bottom) of these two exemplary cells is also shown across all frames. Figure 1*Aii* depicts the percentage of coactive neurons (top) and cell-specific spike activity (raster plot; bottom) across all 120 frames from the WT (left) and Panx1 KO (right) exemplary FOV. In this exemplary FOV, there are more red crosses (spikes from cells participating in a network ensemble) in the Panx1 KO raster plot. Note that total of 27/1044 cells (2.9%) were removed from WT (2.9%) and 66/1155 cells (5.7%) were from Panx1 KO coverslips according to our exclusion criteria (cells exhibiting sustained fluorescence intensity at 90% of maximum or greater). Consistent with this exemplary data, overall, Panx1 KO cultures exhibited a significant increase in network ensembles (Fig. 1*Bi*; *p* = 0.0014^a1^) and number of cells per ensemble (Fig. 1*Bii*; *p* < 0.0001^a2^), as well as a significant increase in core ensembles (coactive groups of neurons active in more than one network ensemble; Fig. 1*Ci*; *p =* 0.0071^b1^). These observed effects on network properties were conserved with or without the excluded cells. We then looked at raw fluorescence intensity values from Fluo-4 am labeled primary cortical neurons and plotted the median (defined as baseline for this analysis) and the difference between the maximum and minimum fluorescence intensity values (Δ*F*, fluorescence intensity range) for each neuron recorded during our imaging sessions. Panx1 KO neurons exhibited a significant increase in the baseline intensity of Ca^2+^ transients (Fig. 1*D*; *p* < 0.0001^c^) and range of fluorescence compared with WT neurons (Fig. 1*E*; *p* < 0.0001^d^). Additionally, we examined cell-type composition and cell viability. WT and Panx1 KO DIV12-13 cortical neuron cultures were composed of highly similar percentages of excitatory neurons (∼80%), inhibitory neurons (∼16%), and astrocytes (2-4%; Fig. 1*F*; *p* = 0.9702^e4,8^, *p* = 0.7500^e5,9^, *p* = 0.1026^e6,10^, respectively). The low percentage of interneurons is consistent with previous data from this developmental time point (Habets et al., 1987; Benson et al., 1994; Frega et al., 2014; Johnson et al., 2015). Similarly, cell viability assessed by the conversion of MTT to formazan (MTT assay) was not significantly different between the two groups (Fig. 1*G*; *p* = 0.9089^f^). Together, these data suggest that Panx1 KO enhances functional connectivity of developing networks cortical neurons.

### Panx1 Is enriched in synaptic compartments

**Figure 2.**
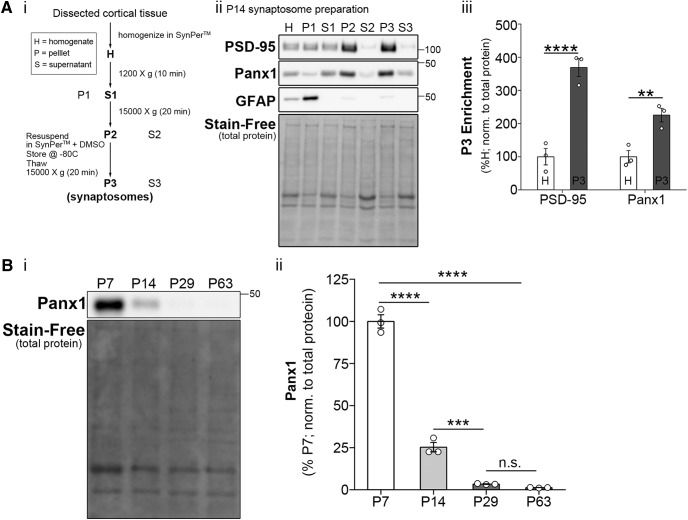
Panx1 is enriched in synaptic compartments. ***A***, Synaptic protein extraction and isolation revealed Panx1 enrichment in cortical synaptic compartments. ***Ai***, Protocol for synaptosome preparation from dissected cortical tissue using SynPer. ***Aii***, Western blot of subcellular fractionations obtained from a P14 WT brain and probed with PSD-95 (top), Panx1 (second panel), and GFAP (third panel), with Stain-Free (total protein) at the bottom, demonstrating enrichment of PSD-95 in the P3 fraction (synaptosomes) and exclusion of GFAP (*negative control*). ***Aiii***, Quantification of Panx1 enrichment in synaptic compartments as determined by higher immunoreactivity in P3 (synaptosomes) relative to homogenate. As expected, PSD-95 was also enriched in P3 (Panx1, *p* = 0.0093^g6,8^; PSD-95, *p* < 0.0001^g5,7^; simple-effect ANOVA with Bonferroni’s multiple-comparison test; *n* = 3 animals; **p < 0.01, ****p < 0.0001). ***B***, Panx1 cortical expression is developmentally down regulated. ***Bi***, Western blot of WT dissected whole cortical tissues from P7-P63 animals, probed with Panx1 (top), and Stain-Free (total protein) at the bottom. ***Bii***, Panx1 expression decreased with age (age: *F*_(3,8)_ = 365.9, *p* < 0.0001^h1^; *n* = 3 animals per group; ****p < 0.0001) with levels markedly dropping from P7 to P14 (*p* < 0.0001^h2^; P14–P29, *p* = 0.0006^h3^; P29–P63, *p* = 0.9604^h4^; one-way ANOVA with Bonferroni’s comparison test; *n* = 3 animals per age group; ***p < 0.001; ****p < 0.0001; n.s., not significant). Data are presented as mean ± SEM.

To confirm expression of Panx1 in synaptic compartments, P14 cortical synaptosome fractions were prepared and validated by enrichment for PSD-95, and exclusion of the astrocyte protein GFAP by Western blotting (Fig. 2*A*). The synaptosome fractions demonstrated specific enrichment of Panx1 (Fig. 2*Aiii*; *p* = 0.0093^g6,8^). Western blot analysis of whole cortical lysates from WT (C57BL/6J) mice revealed a dramatic drop in Panx1 expression from P7 to P14, and further, from P14 to P29 (Fig. 2*Bii*; *p* < 0.0001^g2^, *p* = 0.0006^g3^), consistent with previous reports demonstrating peak Panx1 transcript expression at embryonic day (E)18 followed by a precipitous postnatal decreased (Ray et al., 2005; Vogt et al., 2005).

### Increased PSD-95 and altered postsynaptic receptor expression in Panx1 KO cortical synaptosomes

**Figure 3.**
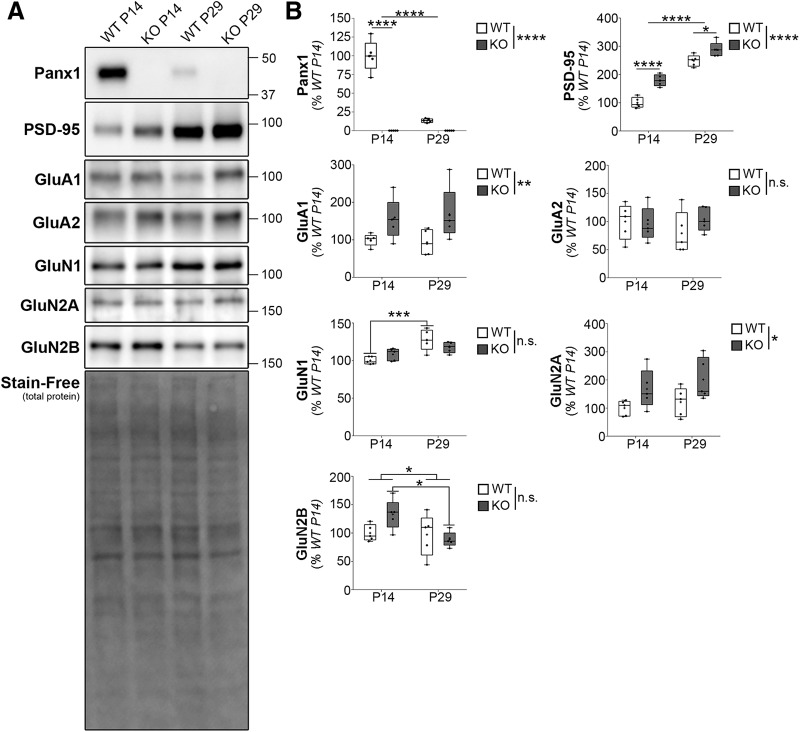
Increased PSD-95 and altered postsynaptic receptor expression in Panx1 KO cortical synaptosomes. ***A***, Representative Western blots of cortical synaptosome preparations from WT and Panx1 KO (P14 and P29) probed for Panx1, PSD-95, and glutamate postsynaptic receptor subunits (GluA1, GluA2, GluN1, GluN2A, GluN2B). The Bio-Rad Stain-Free reagent (bottom) was used to quantify total protein for normalization. Molecular weight markers are indicated in kilodaltons. ***B***, Quantification of protein expression levels of Panx1, PSD-95, and post-synaptic glutamate receptors. Expression levels for each protein were normalized to total protein and expressed as a percentage of WT P14 values; *n* = 5 animals per group analyzed in five independent experiments. Panx1 significantly decreased from P14 to P29 in WT cortical synaptosomes (P14 = 100 ± 9.4%; P29 = 13.4 ± 1.2%, *p* < 0.0001^i3,4,7^; simple effect ANOVA with Bonferroni’s multiple-comparison test; ****p < 0.0001). No Panx1 signal was detected in Panx1 KO cortical synaptosomes**.** PSD-95 significantly increased with age in both WT and Panx1 KO, and was also significantly higher in Panx1 KO relative to WT within age-matched controls (age: *F*_(1,16)_ = 37.4, *p* < 0.0001^j3^; genotype: *F*_(1,6_ = 175.8, *p <* 0.0001^j2^; interaction: *F*_(1,16)_ = 4.2, *p* = 0.0570^j1^; two-way ANOVA with Bonferroni’s multiple-comparison test; WT P14 = 100 ± 8.5%, KO P14 = 179.2 ± 9.1%, *p* < 0.0001^j4^; WT P29 = 248.5 ± 9.0%, KO P29 = 287.9 ± 11.8%, *p* = 0.0220^j5^; *p < 0.05, ****p < 0.0001). GluA1 and GluN2a also exhibited age-matched increases in expression in Panx1 KO cortical synaptosomes (GluA1: genotype, *F*_(1,16)_ = 9.090, WT P14 = 100 ± 7.2%, KO P14 = 155.6 ± 24.4%; WT P29 = 93.42 ± 14.9%, KO P29 = 168.4 ± 31.7%, *p* = 0.0082^k2^; GluN2A: *F*_(1,16)_ = 7.892, WT P14 = 100 ± 12.2%, KO P14 = 167.8 ± 31.20%; WT P29 = 121.4 ± 23.2%, KO P29 = 201.3 ± 33.3%, *p* = 0.0126^n2^); *p < 0.05, **p < 0.01, GluN1 developmental upregulation was more pronounced in the WT group (*p* = 0.0009^m1-8^); ***p < 0.001, whereas GluN2B immunoreactivity in Panx1 KO synaptosomes exhibited a steeper developmental decline at P29 compared to WT (age: *F*_(1,16)_ = 4.547, *p* = 0.0488^o3^; WT P14 = 100 ± 6.5%, WT P29 = 97.1 ± 16.6%, *p* > 0.9999^o4^; KO P14 = 133.1 ± 11.9%, KO P29 = 88.6 ± 5.9%; *p* = 0.0240^o5^; two-way ANOVA with Bonferroni’s multiple-comparison test; *p < 0.05). Data are presented as mean ± SEM. For additional statistical information, see Table 1^i1-o5^.

Similar to the changes observed in Panx1 expression in whole cortex lysates, Panx1 expression in cortical synaptosomes dropped markedly (∼80%) between P14 and P29 (Fig. 3*A*,*B*; *p* < 0.0001^g3,4,7^). Panx1 was not detected in cortical synaptosomes from global Panx1 KO mice (Fig. 3*A*,*B*). Consistent with the rapid development of dendritic spines in the first month of postnatal life (Miller, 1986; Zuo et al., 2005; Romand et al., 2011), expression of PSD-95 increased significantly between P14 and P29 (Fig. 3*B*; *p* < 0.0001^h3^). Somewhat unexpectedly, PSD-95 was further increased in Panx1 KO synaptosomes relative to synaptosomes from age matched WT controls (P14, *p* < 0.0001^h4^; P29, *p* = 0.0220^h5^). These changes were accompanied by significant increases in GluA1 and GluN2A in Panx1 KO synaptosomes (Fig. 3*B*; *p* = 0.0082^i2^ and *p* = 0.0126^l2^, respectively). Interestingly, the developmental increase in GluN1 was more pronounced in WT synaptosomes (*p* = 0.0009^m7^), whereas GluN2B levels in Panx1 KO synaptosomes were higher at P14 and showed a more marked developmental decline at P29 (*p* = 0.0488^o3^, *p* = 0.0240^o5^). Because the elevated PSD-95 levels and altered expression of glutamate postsynaptic receptor subunits in Panx1 KO synaptosomes could result from changes in the number of dendritic spines, we next investigated the impact of Panx1 KO on the density of dendritic spines in cortical neurons *ex vivo*.

### Increased dendritic spine densities in cortical neurons from Panx1 KO mice

**Figure 4.**
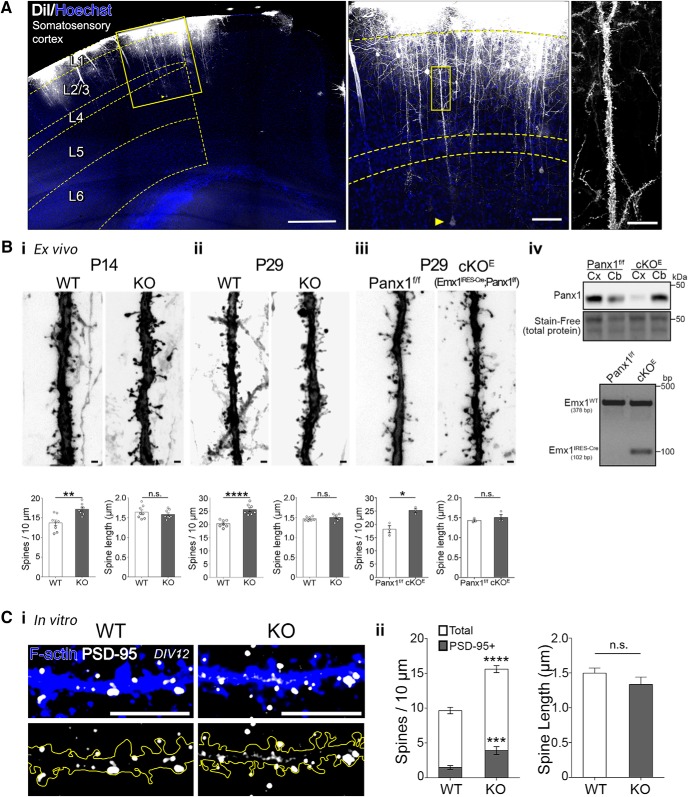
Increased dendritic spine density in Panx1 KO cortical neurons. ***A***, Experimental setup for DiI labeling of apical dendrites of layer 5 somatosensory neurons *ex vivo*. On the left is a representative micrograph of a WT P14 mouse cortex labeled on the pial surface with DiI with an overlay delimiting the somatosensory cortex and cortical layers. A yellow arrow denotes the cell bodies of the layer 5 cortical neuron, shown in the inset; scale bar, 100 µm. On the right, a 100 µm segment of the primary apical dendrite of the cell in the inset, traversing layer 2/3; scale bar, 20 µm. Scale bar, 500 µm. ***B***, Increased dendritic spine density in Panx1 KO cortical neurons. ***Bi***, Representative maximum intensity projections of Panx1 WT (left) and Panx1 KO (right) neurons at P14. Scale bar, 1 µm. Average spine density was significantly higher in Panx1 KO (WT, 13.7 ± 0.7 spines per 10 µm; KO, 17.2 ± 0.5 spines per 10 µm, *p* = 0.0014^p1^; *t*_(14)_ = 3.9, unpaired *t* test, *n* = 8 animals per genotype; **p < 0.01). Average spine length was not significantly different (WT, 1.64 ± 0.06 µm; KO, 1.58 ± 0.04 µm, *p* = 0.4133^p2^; *t*_(14)_ = 0.8, unpaired *t* test, *n* = 8 animals per genotype; n.s., not significant). ***Bii***, At P29. average spine density was significantly higher in Panx1 KO (WT, 20.3 ± 0.5 spines per 10 µm; KO, 25.6 ± 0.8 spines per 10 µm; *t*_(12)_ = 5.8, *p* < 0.0001^q1^, unpaired *t* test, *n* = 7 animals per genotype; ****p < 0.0001). Average spine length was not significantly different (WT, 1.47 ± 0.01 µm; KO, 1.50 ± 0.03 µm, *p* = 0.4274^q2^; *t*_(12)_ = 0.8, unpaired *t* test, *n* = 8 animals per genotype; n.s., not significant). ***Biii***, Similarly, average spine density was significantly higher at P29 in a conditional excitatory neocortical pyramidal cell Panx1 KO (Emx1^IRES-Cre/+^;Panx1^f/f^, Panx1 cKO^E^) compared with Panx1^f/f^ littermate controls (Panx1^f/f^, 18.2 ± 1.4 spines per 10 µm; cKO^E^, 25.3 ± 0.8 spines per 10 µm *t*_(4)_ = 4.6; *p* = 0.0104^r1^, unpaired *t* test, *n* = 3 mice per genotype; *p < 0.05). Average spine length was not significantly different (Panx1^f/f^, 1.43 ± 0.03 µm; cKO^E^, 1.50 ± 0.08 µm, *p* = 0.4326^p2^; *t*_(4)_ = 0.9, unpaired *t* test, *n* = 3 animals per genotype; n.s., not significant). Data are represented as mean ± SEM. ***Biv***, *Top*, representative Western blot of cortical (Cx) and cerebellar (Cb) lysates from control (Panx1^f/f^) and Panx1 cKO^E^ mice. *Bottom*, Genotyping results assaying for the presence of Cre and Emx1 in Panx1^f/f^ and Panx1 cKO^E^. See Methods for more details. ***Ci***, Increased dendritic spine density and PSD-95-positive spine density in cultured cortical neurons at DIV12-14. Representative maximum intensity projections of primary neurite (longest neurite) distal segments from WT and Panx1 KO cultured cortical neurons. Dendritic spines were identified using the phalloidin (F-actin; blue). PSD-95 puncta (white) were quantified (PSD-95+ spines). Scale bar, 10 µm. ***Cii***, Quantification revealed increased mean spine density (WT, 10 ± 0.6 spines per 10 µm; KO, 16 ± 0.5 spines per 10 µm, *t*_(25)_ = 8.4, *p* < 0.0001^s1^; unpaired *t* test, *n* = 10–17 cells from 3 independent cultures; ****p < 0.0001), and increased density of PSD-95-positive spines in Panx1 KO cultured cortical neurons (WT, 1.5 ± 0.3 spines per 10 µm; KO, 3.9 ± 0.6 spines per 10 µm, *t*_(25)_ = 4.2, *p* = 0.003^s2^; unpaired *t* test, *n* = 10–17 neurons from 3 independent cultures; ***p < 0.001). Spine length was not different between groups (*p* = 0.2047^s3^). Data are presented as mean ± SEM.

Based on our finding that synaptosomal PSD-95 expression was selectively increased in age-matched Panx1 KO cortical synaptosomes, we tested the hypothesis that Panx1 regulates dendritic spine development. We used the fluorescent lipophilic dye, DiI, which allows for relatively sparse labeling of somatosensory layer 5 pyramidal neurons (Fig. 4*A*). As predicted, spine densities from the apical dendritic tuft of layer 5 pyramidal neurons were significantly higher in Panx1 global KO mice than age matched WT controls at both P14 and P29 (Fig. 4*Bi*,*Bii*; P14, *p* = 0.0014^p1^; P29, *p* < 0.0001^q1^). These changes were consistent with the increased synaptosome PSD-95 expression levels observed at P14 and P29. Next, because Panx1 has also been detected in astrocytes (Huang et al., 2007; Iglesias et al., 2009), microglia (Burma et al., 2017), and several vascular system cell types (Begandt et al., 2017) in various contexts, we also generated a conditional glutamatergic neuron-specific (Emx-1^IRES-Cre^;Panx1^f/f^) Panx1 KO (Panx1cKO^E^). Consistent with our results from the global Panx1 KO, spine densities in Panx1cKO^E^ were significantly higher than in Panx1^f/f^ controls (Fig. 4*Biii*; *p* = 0.0104^r1^). The Cre-based recombination in the Emx1-expressing lineage begins as early as E10.5 (Gorski et al., 2002). Western blot analysis (Fig. 4*Biv*) of Panx1cKO^E^ and control (Panx1^f/f^) cortical (Cx) and cerebellar (Cb) lysates demonstrated marked reduction of Panx1 immunoreactivity in Panx1cKO^E^ cortical lysates, confirming Panx1 KO in cortical excitatory neurons (comprising the majority of cortical tissue). Notably, mean spine lengths were not significantly different in either Panx1 KO line, suggesting the additional spines do not represent abnormally long spines or filopodia (Miller and Peters, 1981; Ziv and Smith, 1996; Zuo et al., 2005; for review, see Sala and Segal, 2014). Panx1 KO primary cortical neurons grown in culture for DIV12-14 exhibited significantly higher densities of dendritic protrusions resembling dendritic spines (Fig. 4*C*). A similar proportion of dendritic protrusions co-localized with PSD-95 in both WT and Panx1 KO cortical neurons and spine lengths were not significantly different between groups. Together these results suggest that deletion of Panx1 in glutamatergic cortical neurons increases spine density in a cell-autonomous way.

## Discussion

To our knowledge, this is the first study connecting Panx1 to the structural development of dendritic spines. We observed similar spine lengths and proportions of spines expressing PSD-95 in WT and Panx1 KO cortical neurons, suggesting Panx1 KO does not simply induce a selective proliferation of immature spines, but rather increases the number of spines with very similar properties to those found in WT cortical neurons. Our results suggest that this increase in dendritic spine density underlies the larger number of network ensembles observed in Panx1 KO cortical cultures. These findings are consistent with recent evidence demonstrating that incorporation of a cell into a network ensemble requires the development of spines and synapses (Jung and Herms, 2014; for review, see Hoshiba et al., 2017; Frank et al., 2018). While cortical cultures, in which we performed our network analysis, are known to contain abundant autaptic connections, these are also highly abundant within the developing rodent neocortex. Lübke et al. (1996) reported that autaptic contacts are found in most layer 5 cortical neurons *in situ* in the developing rat neocortex (92% of all coupled neurons; 80% of all cells analyzed). Among others, a recent report from Yin et al. (2018) confirmed that autapses occur in layer 5 pyramidal neurons in developing mouse prefrontal cortex and human frontal lobe (acute brain slices) that persist into adulthood, promoting neuronal responsiveness, burst firing and coincidence detection. Thus, not only might Panx1 KO impact autapses in culture but also potentially *in vivo*. It is also important to note that our understanding of the contribution of spine development and synaptic strengthening to spontaneous network development is still relatively limited and our experiments did not address whether the ∼20–30% increases in spine density we observed *ex vivo* and *in vitro* equated directly to 20–30% increases in the number of synapses. Finally, given that not all synapses/cells are recruited to shape the development of neuronal network ensembles, and because our current understanding of the recruitment process is limited (Hoshiba et al., 2017), in the absence of more sophisticated methodology, we are unable to predict which Panx1 KO cells might be engaged in enhanced coupling. Moreover, rescue experiments in which Panx1 is re-expressed in control and Panx1 KO neurons are now needed to determine whether the role of Panx1 in regulating spine formation and network ensembles is direct.

The current study expands on previous findings relating to synaptic plasticity in Panx1 KO mice by targeting a different region of the brain, the cortex, and by focusing on potential developmental contributions. Previous studies looked primmarily at the CA1 region of the hippocampus at 1 month of age or older (Prochnow et al., 2012; Ardiles et al., 2014). These studies showed increased CA1 long-term potentiation (Prochnow et al., 2009; Ardiles et al., 2014; Gajardo et al., 2018) as well as a reduction in LTD (Ardiles et al., 2014) associated with Panx1 KO; albeit, these effects were observed uniquely in adult animals [3 months for the Prochnow et al. (2009) study, 9–12 months for the Ardiles et al. (2014) study]. Ardiles et al. (2014) found that hippocampal Panx1 expression levels were greatly reduced between young (1 month) and older (9–12-month-old) animals, which is consistent with our findings over our earlier age range, and suggest that the decline in Panx1 levels begins in the early postnatal period and continues on with increasing age. More recent work showed that Panx1 channels are strongly active under ictal conditions in human brain cortical tissue from epilepsy patients and in the CA1 region of the hippocampus following kainic acid seizure induction (Dossi et al., 2018), suggesting that in pathologic conditions Panx1 is positively correlated with excitability; although the mechanistic underpinnings, such as the possible interneuronal or glial contributions to this effect, have not yet been fully resolved. Relatedly, because Panx1 has been detected in multiple cell types and has been associated with cell death processes (for review, see Sandilos and Bayliss, 2012; Thompson, 2015; Swayne and Boyce, 2017), we analyzed the cellular composition and viability of our cortical cultures. WT and Panx1 KO cultures were comprised of similar percentages of excitatory neurons, inhibitory neurons and astrocytes. The majority of cells in both WT and Panx1 KO cultures were excitatory neurons (∼80%). Inhibitory neurons, although less abundant (∼16% in our cultures for both WT and Panx1 KO), play an important role in shaping cortical networks (Lu et al., 2017). Panx1 has been detected in both excitatory and inhibitory neurons (Ray et al., 2005; Vogt et al., 2005; Zoidl et al., 2007), and this is consistent with our Western blot data from Panx1cKO^E^ (glutamatergic-specific Panx1 KO) and control (Panx1^f/f^) lysates. Cortical control (Panx1^f/f^) lysates exhibited a minor residual Panx1 immunoreactivity, which likely reflects expression in inhibitory neurons. While together our results suggest that the impact of Panx1 KO on dendritic spine formation is cell-autonomous (glutamatergic neurons), a potential contribution of inhibitory neuron Panx1 to network ensemble development remains to be determined.

The mechanisms governing spine formation and plasticity are poorly understood (for review, see Yoshihara et al., 2009; Murakoshi and Yasuda, 2012), and are developmental age- and brain region-specific, making it difficult to directly compare these previous studies with our own. For example the major chloride extruder in neurons, KCC2, differentially regulates brain-derived neurotrophic factor (BDNF)-dependent dendritic spine development in CA1 and somatosensory neurons during the first postnatal week (Awad et al., 2018). This study suggests molecular mechanisms of dendritic spine formation in different brain regions might not be completely generalizable. Moreover, mounting evidence implicates Panx1 as a possible chloride permeable channel (Ma et al., 2012; Nomura et al., 2017) and thus this differential regulation and of a chloride extruder could directly impact on Panx1 in spine formation; although it remains to be confirmed whether Panx1 channel function itself is implicated in the regulation of spine development. Further, BDNF, which regulates the brain region-specific effect of KCC2 (Awad et al., 2018), is expressed at higher levels in the hippocampus than in the cortex (Awad et al., 2018), suggesting fundamental differences in baseline levels of key molecular effectors of synaptic plasticity.

Activity-driven spine stabilization requires PSD-95 (Ehrlich et al., 2007); PSD-95 is more frequently detected at stable rather than transient synaptic contacts (Taft and Turrigiano, 2014), and its overexpression increases synaptic contact stability (El-Husseini et al., 2000). Moreover, most spines lacking a PSD do not persist >1 d, and further, a reduction in PSD-95 precedes spine loss (Cane et al., 2014). BDNF-dependent PSD-95 delivery into spines requires tubulin polymerization (Hu et al., 2011). Microtubule spine invasion is thought to be necessary for kinesin-based transport of cargo required for spine stabilization (for review, see Dent, 2017); although the mechanisms regulating this process are still relatively unknown. A recent report described an interaction between Panx1 and Crmp2 (Xu et al., 2018), a protein that regulates microtubule stabilization and elongation (Fukata et al., 2002; Niwa et al., 2017). Block of Panx1 with probenecid reduces the Panx1-Crmp2 interaction and concomitantly results in increased microtubule stability and enhance neurite outgrowth (Xu et al., 2018). Moreover, deletion of Crmp2 reduces dendritic spine density (Zhang et al., 2016). Of note, synaptosome fractions of WT and Panx1 KO cortices at P14 and P29 exhibited similar Crmp2 protein levels (data not shown). Together these findings suggest a working model in which Panx1 sequesters Crmp2 by physical interaction until a specific cue, possibility a local elevation in extracellular ATP (Boyce et al., 2015; Boyce and Swayne, 2017), triggers Panx1 downregulation in the spine/shaft and release of Crmp2 to facilitate microtubule elongation, invasion and delivery of PSD-95 into the spine thereby facilitating associated downstream molecular events associated with spine growth and stability. The role of Panx1-interacting proteins in Panx1 regulation of spine development will be the focus of future studies.

Altogether, our novel findings presented here have implications for understanding neurodevelopment and diseases involving changes in spines. Alterations in dendritic spine densities have been described in a variety of neuropsychiatric disorders (Swann et al., 2000; Kulkarni and Firestein, 2012; Forrest et al., 2018). Of note, a recent study identified a human *PANX1* variant with multi-organ developmental abnormalities associated with marked intellectual disability (Shao et al., 2016). Moreover, single nucleotide polymorphisms affecting Panx1 expression levels have been implicated in autism spectrum disorder (Davis et al., 2012). Therefore, understanding the developmental role of Panx1 could provide important insights into variations in normal brain development as well as risk of neuropsychiatric disease.

## Synthesis

Reviewing Editor: Katalin Toth, Universite Laval Decisions are customarily a result of the Reviewing Editor and the peer reviewers coming together and discussing their recommendations until a consensus is reached. When revisions are invited, a fact-based synthesis statement explaining their decision and outlining what is needed to prepare a revision will be listed below. The following reviewer(s) agreed to reveal their identity: Roger Thompson, Karun Singh.

Both reviewers thought that the present study provides novel and important information about the function of Panx1 regarding its role in neuronal excitability and spine number. While the reports are generally positive both reviewers raised several issues that needs to be addressed.

The authors should comment on the possibility that autaptic synapses might be afftected in the Panx1 KO. Culutred neurons have a propensity to fire together or appear to because of autaptic connections. Is it possible that knocking out pannexin is increasing autapses and this is an artifact of culture?

It would be very important to carefully address Point 2 of Rev.#1. This is a really critical point and ideally it would be addressed experimentally. If possible, few electric recordings (that have appropriate temporal resolution to investigate AP firing) should help to resolve this problem. Parallel recording from at least 2 cells would provide indisputable data regarding ensemble formation.

Rev. #2 suggest an important experiment in his/her Point 1. Wille this is a great idea, this would require more work than it is reasonable to expect in the frame of this study. However, the reviewer's concern should be discussed in the manuscript.

Rev.#1.

Manuscript eN-NWR-0503-18 presents an interesting description of the role of pannexin-1 knockout on cultured neuronal excitability and development of dendritic spine numbers. This was achieved with several experimental approaches involving constitutive and conditional knockout mouse models. The main conclusion is that knockout of Panx1 increases spine numbers in cortical neurons and this correlates with augmented activity and more network ensembles (i.e. about double the number of cultured neurons appear to fire together). The results are correlative, but when considered with other work in the field a model of the mechanism emerges that is presented in the Discussion. A visual abstract would have helped with the communication of this concept. I have some concerns that need to be addressed.

major:

1) Figure 1 shows the Fluo-4 based imaging of calcium in cultured cortical neurons. These are presumably mixed cultures of excitatory, inhibitory and glial cell populations. Thus, it is not clear from the images what types of neurons are showing increased activity.It is presumably excitatory glutamatergic cells based on the focus of the paper, but this is not demonstrated. What portion of these are GABAergic interneurons versus glutamatergic excitatory cells? Since pannexin is expressed in populations of interneurons and interneurons are important in controlling synchronous firing (i.e. ensembles) the authors should make an attempt to identify the cell types. Performing similar calcium imaging experiments in cultures from their conditional KO mice could provide important insight?

2) I have a technical concern about the calcium imaging. Images were collected every 5s (0.2 hZ) which is much much slower than the timing of an action potential / lifetime of normal calcium levels. To my mind this would greatly underestimate the ability to detect neurons firing together. What magnitude of error is this introducing into the ensemble analysis? Could it be that the neurons are just more excitable and therefore the probability of seeing them fire together is increased by Panx1 KO, giving the appearance of ensemble formation but that the cells themselves are not ‘driving’ each other?

2) the Emx-1:Panx1fl/fl mice are new as far as I can find. Please provide data showing the extent of pannexin1 knockout, particularly in cortical neurons used in the study.

minor:

1) the authors also measured GluN2b levels but I found no mention of it in the text.

Rev.#2

In the eNeuro submission “Pannexin 1 regulates network ensembles and dendritic spine development in cortical neurons”, the authors describe the function of Pannexin 1 in regulating dendritic spine density and communication between cortical neurons using calcium imaging. By using the proportion of neurons with high fluorescence per frame, they show strong evidence that loss of Panx1 increases overall single and global neuron activity. Furthermore, they provide strong evidence that Panx1 is enriched at the post-synaptic density early in development and that loss of Panx1 results in reduced synaptic number on excitatory cortical neurons, without altering the overall maturity of neurons. This is a very nice study, with solid data, but there is room to further clarify certain discussion points and to improve the data.

1. The KO data is lacking a rescue experiment to show that re-expression of Panx1 can recue either the calcium imaging data and/or dendritic spine abnormalities. This is critical to show that the increase in spine density is directly associated with the increase in network ensembles due to loss of Panx1.

2. The authors should justify why they chose the time point DIV12 for their calcium imaging studies. At this time point, there is minimal synaptic connections between cultured neurons, which increases over the next 1-2 weeks in vitro. Have they tried older cultures and is the increased ensembles still present?

3. Is Panx1 involved in cell death, and can the authors rule out that cell death, is not playing a role in the calcium imaging or dendritic spine phenotypes observed in Panx1 KO's?

4. The authors mention that CRMP2 is an interactor of Panx1. Are there changes in the levels or localization of CRMP2 in the Panx1 KO neurons for the phenotypes they observed?

Small comments and suggestions:

- Line 306: there is no 2Biii in Figure 2

- Line 376: the authors show that decreased panx1 results in increased neuron activity, however in the discussion they state that panx1 positively correlates with excitation?

- Line 342-346: the in vitro cultures do show higher dendritic spine density, however this does not provide evidence that loss of Panx1 does not increase immature spines in vitro. The length of spines could also be shown for these experiments; also, they could also quantify mature spines to see what is the impact on Panx1 KO neurons

- Line 633: there is no Di in Figure 1
